# The current and possible future role of 3D modelling within oesophagogastric surgery: a scoping review

**DOI:** 10.1007/s00464-022-09176-z

**Published:** 2022-03-11

**Authors:** Henry Robb, Gemma Scrimgeour, Piers Boshier, Anna Przedlacka, Svetlana Balyasnikova, Gina Brown, Fernando Bello, Christos Kontovounisios

**Affiliations:** 1 Imperial College Healthcare NHS Trust, London, UK; 2 Imperial College London, London, UK; 3 The Royal Marsden NHS Foundation Trust, London, UK; 4 Chelsea Westminster NHS Foundation Trust, London, UK

**Keywords:** Computer-generated 3D imaging, Gastrointestinal diseases, General surgery, 3D printing, Virtual reality, Augmented reality

## Abstract

**Background:**

3D reconstruction technology could revolutionise medicine. Within surgery, 3D reconstruction has a growing role in operative planning and procedures, surgical education and training as well as patient engagement. Whilst virtual and 3D printed models are already used in many surgical specialities, oesophagogastric surgery has been slow in their adoption. Therefore, the authors undertook a scoping review to clarify the current and future roles of 3D modelling in oesophagogastric surgery, highlighting gaps in the literature and implications for future research.

**Methods:**

A scoping review protocol was developed using a comprehensive search strategy based on internationally accepted guidelines and tailored for key databases (MEDLINE, Embase, Elsevier Scopus and ISI Web of Science). This is available through the Open Science Framework (osf.io/ta789) and was published in a peer-reviewed journal. Included studies underwent screening and full text review before inclusion. A thematic analysis was performed using pre-determined overarching themes: (i) surgical training and education, (ii) patient education and engagement, and (iii) operative planning and surgical practice. Where applicable, subthemes were generated.

**Results:**

A total of 56 papers were included. Most research was low-grade with 88% (*n* = 49) of publications at or below level III evidence. No randomised control trials or systematic reviews were found. Most literature (86%, *n* = 48) explored 3D reconstruction within operative planning. These were divided into subthemes of pre-operative (77%, *n* = 43) and intra-operative guidance (9%, *n* = 5). Few papers reported on surgical training and education (14%, *n* = 8), and were evenly subcategorised into virtual reality simulation (7%, *n* = 4) and anatomical teaching (7%, *n* = 4). No studies utilising 3D modelling for patient engagement and education were found.

**Conclusion:**

The use of 3D reconstruction is in its infancy in oesophagogastric surgery. The quality of evidence is low and key themes, such as patient engagement and education, remain unexplored. Without high quality research evaluating the application and benefits of 3D modelling, oesophagogastric surgery may be left behind.

**Supplementary Information:**

The online version contains supplementary material available at 10.1007/s00464-022-09176-z.

## Background

Since three-dimensional (3D) anatomical models were first created from two-dimensional (2D) computational tomography (CT) images in 1979, 3D reconstruction has become increasingly common in medicine due to rapid technological advances [1–3]. 3D reconstructions have various applications, including the manufacture of 3D printed (3DP) models and Virtual Reality (VR) simulators [4, 5].

**Fig. 1:**
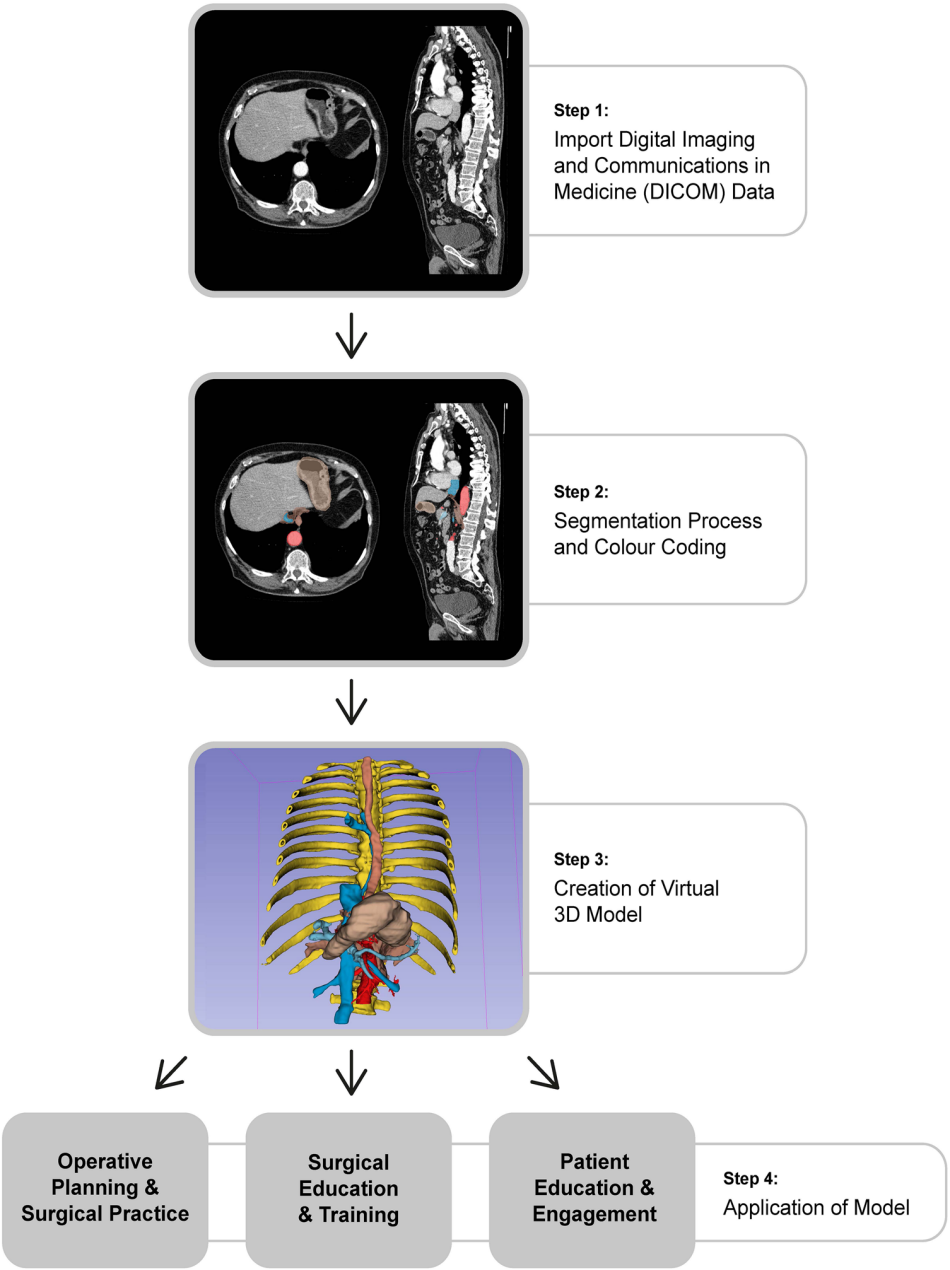
Creation of a virtual reality 3D model and its application

In surgery, 3D reconstruction has been used for operative planning, surgical training, and patient engagement (Fig. 1). Using 3D reconstructions, complex anatomical relationships can be clearly visualised to help guide surgical decision making [6]. Shen et al. demonstrated that pre-operative 3D reconstructions can improve surgical outcomes and reduce complication rates [7]. As the traditional apprenticeship model of surgical training becomes incompatible with modern practice and working patterns, 3D reconstructive techniques may play an increasing role in surgical training. Creating VR simulators with realistic haptic and stereoscopic feedback has been shown to help junior surgeons develop their skills in a safe environment [8, 9]. 3D reconstructions, whether virtual or 3DP, enhance patient education when compared to standard imaging across a range of specialities [10, 11]. Early studies show that personalised 3D models help individuals gain greater insight into their disease and this improves shared decision-making [12]. It is likely 3D reconstruction will become commonplace in surgical practice and benefit both patients and clinicians.

As a subspecialty, general surgery has been slow in the adoption of 3D reconstruction [13, 14]. This may reflect both limited resource availability as well as image-related, organ-specific complexities. It is generally easier to reconstruct defined structures such as bone or vasculature compared to distensible hollow viscera like the stomach [15, 16]. However, as technological capabilities improve and costs decline [17], 3D reconstruction will become more accessible for use in routine oesophagogastric surgical practices. For the purposes of this review, oesophagogastric surgery is considered to include surgery involving the stomach and oesophagus in benign and malignant states. This will include bariatric surgery.

Herein the authors outline the findings of a scoping review that aims to help clarify current and future roles for 3D modelling in oesophagogastric surgery, highlighting gaps in the literature as well as implications for future practice and research [18, 19].

## Methods

### Protocol

A scoping review protocol, based on internationally accepted guidelines developed by Arksey and O’Malley and later refined by Levac et al. and the Joanna Briggs Institute [20–22], was developed. The authors’ protocol is available via the Open Science Framework (osf.io/ta789) and has been published in a peer-reviewed journal [23]. As the study progressed, minor changes were made to the a priori protocol, as is accepted practice when undertaking scoping reviews [24]. Each amendment was discussed and agreed by the investigatory team prior to its adoption. Since the creation of the scoping review protocol, the title of the study has changed from ‘upper gastrointestinal surgery’ to ‘oesophagogastric surgery’ to clarify the exclusion of hepatobiliary surgery. The review report has been written following the Preferred Reporting Items for Systematic reviews and Meta-analyses extension for Scoping Review (PRISMA-ScR) Checklist [25].

### Search strategy

Working with a specialist medical librarian the lead investigator (HR) developed a comprehensive search strategy tailored for each key database using keywords, thesauri terms and Boolean Operators (Appendix 1). MEDLINE, Embase, Elsevier Scopus and ISI Web of Science were searched from their inception to 1/6/2020. Additional grey literature was identified using OpenGrey and Grey Literature Report. The reference lists from selected studies were reviewed by hand using a ‘snowball search’ methodology to identify additional relevant literature.

### Study selection

A two-stage screening process using ‘title and abstract screening’ and ‘full text review’ was performed by two independent reviewers (HR and GS). Any disagreement was resolved by discussion and if required, a third reviewer (CK) provided a decisive vote. This process was undertaken using the Covidence systematic review software [26]. Full text articles were included if they reported the use of 3D modelling within the setting of oesophagogastric surgery, specifically focussing on the stomach and oesophagus, in health, benign and malignant states. Only studies published in the English language were included. Studies were excluded if they pertained to 3D modelling outwith oesophagogastric surgery, studied paediatric populations, or were not published in peer-reviewed literature. Additional exclusion criteria used after the publication of the protocol included non-human or animal-based research (Appendix 2). As 3D modelling is an emerging technology it was anticipated selected studies would have a comparatively low evidence grade. A methodological quality analysis for exclusion was therefore not performed, as this may have significantly restricted the number of studies evaluated.

### Data extraction

After study selection, two independent reviewers (HR and GS) extracted key study characteristics using a data charting tool to allow both quantitative and narrative assessment. Data extracted included author(s), year of publication, origin of study, study aims, study population, sample size, design, and main findings. To perform a qualitative thematic analysis, selected studies were reviewed and assigned to one of the three pre-determined overarching themes: (i) surgical training and education, (ii) patient education and engagement, and (iii) operative planning and surgical practice. Where applicable, subthemes were generated to enhance thematic assessment. These were then combined into a study summary table which included author(s), title, year of publication, imaging modality, software used, study design, form of 3D modelling and theme explored (Appendix 3).

## Results-quantitative assessment

### Included studies

**Fig. 2:**
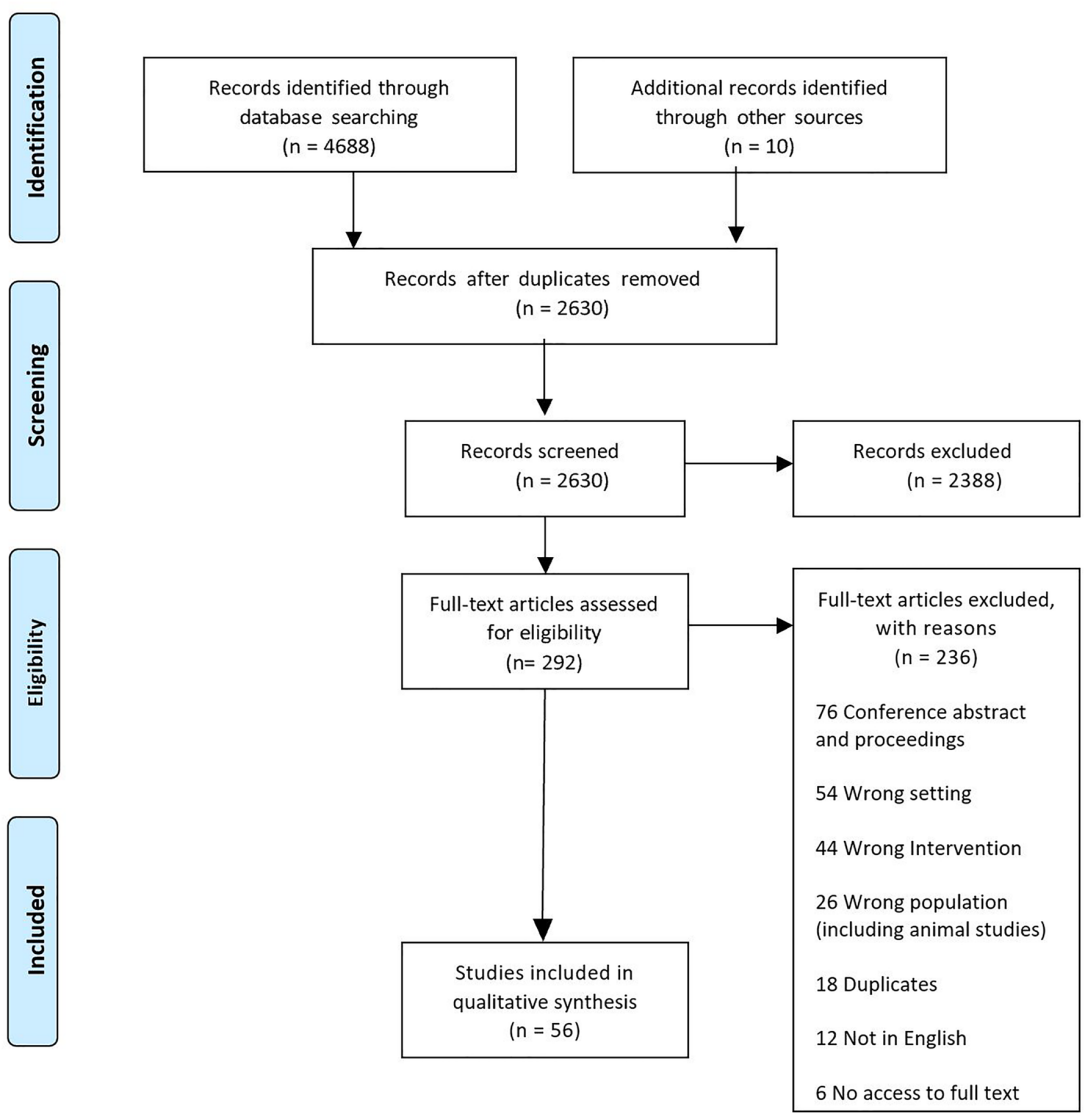
PRIMSA flow diagram for study identification, screening and inclusion

The search yielded 4688 results. Snowball searching and grey literature searches later added a further 10 sources. After duplicate removal, 2630 sources underwent text and abstract screening (Fig. 2 [27]). The full text of 292 sources were reviewed and ultimately 56 papers were included for data extraction (Appendix 3 [28–83]). The most frequent reasons for exclusion were conference abstracts and proceedings without publication in a peer-reviewed journal (*n* = 76), the wrong setting (*n* = 54) and wrong intervention (*n* = 44).

### Study characteristics

Significant heterogeneity in study design, image processing methods, technologies utilised, statistical analysis and outcomes measured was observed. Evidence reported within the majority of included studies was classified as low-grade, with 88% (*n* = 49) of publications falling at or below level III evidence [84]. Only 7 studies met level II evidence criteria and no studies met level I evidence criteria. There were no randomised-control trials (RCTs) of 3D modelling in oesophagogastric surgery. Frequently used study designs included Case Reports and Case Series (21%, *n* = 12), Non-Randomised Controlled Experimental Studies (18%, *n* = 10) and Basic Research studies (18%, *n* = 10). Most of the research, 75% (*n* = 42), originated in East Asia, with South Korea, Japan and China having the highest output. Only 13% (*n* = 7) of published work originated in Europe. All studies were published during or after 2003 and used either virtual or augmented reality. Only 5% (*n* = 3) used 3D printing.

The software used, the segmentation methodology and the rendering technique were inconsistently reported throughout the included texts. The 3D reconstruction software used was not stated in 30% (*n* = 17) of studies. In the remaining literature, 23 different software programmes were described with Zio Software Inc (11%, *n* = 6) the most frequently used. The rendering technique was not stated in 52% (*n* = 29) of included studies and the segmentation methodology was not described 73% (*n* = 41) of studies. However, when stated, volume rendering (41%, *n* = 23) was most frequently described. Predominantly, automatic (5%, *n* = 3) or a mix of automatic and manual segmentation (11%, *n* = 6) methodologies were used.

### Thematic analysis of included studies

The majority (86%, *n* = 48) of studies explored the role of 3D modelling within operative planning and surgical practice. Subtheme analysis showed the most common application of 3D reconstruction was for pre-operative guidance (77%, *n* = 43) with only 5 papers (9%) investigating the potential impact of 3D modelling on intraoperative guidance during oesophagogastric surgery. A small number of studies (14%, *n* = 8) reported on surgical training and education, and subtheme analysis showed these were evenly divided between Virtual Reality Simulation and Anatomical Teaching. No studies utilised 3D modelling for patient engagement and education. A summary of key included papers is provided in Table 1.

**Table 1:** Characteristics and themes of key included studies

Author, Year	Study design	Origin	Virtual reality (VR) and/or 3D printing (3DP)	Main theme	Subtheme
Lewis, (2012)	Non-randomised uncontrolled experimental study	UK	VR	Surgical education and training	VR simulation
Giannotti, (2014)	Non-randomised uncontrolled experimental study	Italy	VR	Surgical education and training	VR simulation
Choi, (2009)	Basic research	South Korea	VR	Surgical education and training	VR simulation
Kavic, (2006)	Basic research	USA	VR	Surgical education and training	Anatomical teaching
Shin, (2009)	Basic research	South Korea	VR	Surgical education and training	Anatomical teaching
Kwon, (2015)	Basic research	South Korea	VR	Surgical education and training	Anatomical teaching
Wu, (2013)	Basic research	China	VR	Surgical education and training	Anatomical teaching
Usui, (2005)	Non-randomised uncontrolled experimental study	Japan	VR	Operative planning and surgical practice	Pre-operative guidance
Matsuki, (2004)	Non-randomised controlled experimental study	Japan	VR	Operative planning and surgical practice	Pre-operative guidance
Zhu, (2018)	Retrospective cohort study	South Korea	VR	Operative planning and surgical practice	Pre-operative guidance
Sunagawa and Kinoshita, (2017)	Case Series	Japan	VR	Operative planning and surgical practice	Pre-operative guidance
Wang, (2014)	Retrospective Case–Control Study	China	VR	Operative planning and surgical practice	Pre-operative guidance
Kinoshita, (2016)	Retrospective cohort study	Japan	VR	Operative planning and surgical practice	Pre-operative guidance
Cai, (2018)	Non-randomised controlled experimental study	China	VR	Operative planning and surgical practice	Pre-operative guidance
Onbaş, (2006)	Non-randomised controlled experimental study	Turkey	VR	Operative planning and surgical practice	Pre-operative guidance
Choi, (2014)	Review	South Korea	VR	Operative planning and surgical practice	Pre-operative guidance
Mamede, (2007)	Non-randomised controlled experimental study	Japan	VR	Operative planning and surgical practice	Pre-operative guidance
Alfieri, (2015)	Non-randomised controlled experimental study	Italy	VR	Operative planning and surgical practice	Pre-operative guidance
Kim, (2005)	Review	South Korea	VR	Operative planning and surgical practice	Pre-operative guidance
Marano, (2019)	Case report	Italy	3DP and VR	Operative planning and surgical practice	Intra-operative guidance
Ye, (2020)	Case report	China	3DP	Operative planning and surgical practice	Intra-operative guidance
Dickinson, (2015)	Case Series	USA	3DP and VR	Operative planning and surgical practice	Intra-operative guidance
Sato, (2020)	Case report	Japan	VR	Operative planning and surgical practice	Intra-operative guidance
Kim, (2013)	Prospective Cohort Study	South Korea	VR	Operative planning and surgical practice	Intra-operative guidance

## Results-narrative summary

### Operative planning and surgical practice

#### Pre-operative guidance

Operative planning and surgical practice is the most populated category in this review, with pre-operative guidance the most researched subtheme. Almost a third of studies in this scoping review looked at virtual oesophagogastric vascular reconstruction (29%, *n* = 16) and its role in operative planning [36–51]. Although these papers all conclude that there is a benefit to generating a ‘vascular roadmap’ for surgical guidance, particularly for identifying anatomical variants for laparoscopic surgery, few provide quantitative evidence of improved patient outcome. Key exceptions include the work by Wang et al. [49] and Kinoshita et al. [51]. Wang et al. found that pre-operative vascular reconstructions using 3D reconstruction significantly reduced operative time (19.70 ± 5.59 min vs 24.47 ± 9.98 min; *p* = 0.001) and blood loss at splenic hilum (13.62 ± 4.50 mL vs 17.92 ± 9.08 mL; *p* = 0.001) compared to no 3D reconstruction during laparoscopic total gastrectomy with spleen-preserving splenic lymph node dissection. However, Kinoshita et al. [51] found no significant difference in operative time, blood loss or complication rate in 3D reconstruction versus no 3D reconstruction but did identify significantly higher lymph node retrieval at station 10 and concluded that the quality of surgery was improved. All these studies offer low-grade evidence, that is often retrospective, and there are no RCTs demonstrating improvements to patient outcomes.

Beyond virtual vascular reconstructions, the review identified several (18%, *n* = 10) innovative case reports and basic research using 3D reconstruction techniques to model atypical or challenging anatomy for pre-operative planning [53–62]. For example, Takanami et al. [58] and Kato et al. [59] describe methods to virtually visualise the thoracic duct prior to oesophagectomy. There is also a growing body of work using 3D reconstruction to assess bariatric surgical patients and plan future surgical intervention [60, 61]. Whilst these novel techniques demonstrate various applications for 3D modelling, they are early basic research and do not show quantitative improvements to patient care.

The remaining literature on 3D modelling for pre-operative guidance focused on radiological assessment of malignancy (29%, *n* = 16) [63–78]. The review identified 10 papers, describing the contribution 3D reconstruction has made to gastric cancer pre-operative staging [64, 66–69, 71–73, 75, 76]. Overall, this work describes how the advancement of multidetector CT (MDCT) in conjunction with 3D modelling techniques has significantly improved the ‘T’ staging of gastric cancer, especially in early gastric cancers. Despite these technological innovations, lymph node staging remains a challenge in gastric malignancy.

For the pre-operative radiological assessment and staging of oesophageal cancer, only 4 papers (7%, *n* = 4) were identified [63, 65, 70, 74]. Both Onbas et al. and Cai et al. conclude virtual CT 3D reconstruction is an accurate, safe, and effective tool in the staging of oesophageal cancer [63, 65]. Cai et al. also states that 3D reconstruction may have a role in assessing pathological response following chemo- and radiotherapy [63]. Whilst this topic is explored by Alfieri et al. and Mamede et al., both are clear further research is necessary [70, 74].

#### Intra-operative guidance

The scoping review identified 5 papers exploring 3D reconstruction for intra-operative guidance (9%, *n* = 5). This includes 2 studies describing the utility of 3D virtual reconstructions as guidance during surgery. The remaining 3 articles, consists of case reports and case series, detailing the application of 3D printed models for operative guidance.

The literature on virtual 3D reconstructions for intra-operative guidance primarily relates to vascular anatomy. In a prospective observational study, vascular reconstructions guided surgeons during robotic gastrectomy with lymphadenectomy in gastric cancer patients [83]. The study demonstrated it was feasible to accurately generate and use individualised vascular roadmaps to prevent vascular injury with minimal (15 minute) increases in operating time. However, this technique required a surgically trained radiologist to orientate the reconstruction throughout the operation. Similarly, Sato et al. used Augmented Reality through the HoloLens system to safely manage aberrant vascular anatomy during a thoracoscopic esophagectomy [82]. Both studies suggest these techniques offer significant advantages to novice surgeons but highlight a need to validate these techniques with respect to surgical outcomes.

The work by Marano et al., Dickenson et al. and Ye et al. describe the subjective clinical benefit 3D printed models have for pre-operative planning and intra-operative guidance [79–81]. They encompass its application to robotic, endoscopic, laparoscopic, and open gastro-oesophageal surgery for intricate and complex anatomy. Each paper highlights the improved visual and tactile information provided to the surgical team by 3D printed models in comparison to virtual reconstructions or standard 2D imaging. This information allows the surgeons not only to optimise the surgical approach and rehearse the procedure but then acts as a reference during surgery by showing the relationships of key anatomical structures. Each case was completed without significant complications, which the authors conclude is due to the unique advantages of personalised 3D printed models. However, Marano et al. highlight the considerable production time and high expense of the printed model. No other paper described the cost and time commitments of their technique. No quantitative benefit was demonstrated in these studies.

Although these papers suggest 3D reconstruction and 3D printing could potentially allow for personalised and safer operating, this has not been quantified and questions remain about the high cost and time required.

### Surgical education and training

We identified 8 papers (14%, *n* = 8) under the theme of surgical education and training that use 3D reconstruction for oesophagogastric surgery. These are subcategorised into Virtual Reality (VR) simulation and anatomical teaching.

The review identified 4 papers (7%, *n* = 4) studying VR simulation for oesophagogastric surgical training. The laboratory research by Choi et al. describes the generation of a graphic and haptic VR model of the oesophagus for simulation training. This technology paper describes the creation of the oesophageal 3D model but does not validate it in the context of surgical training. The remaining research on this topic relates to bariatric surgical training. One paper describes the successful creation and validation of a novel VR laparoscopic adjustable gastric band simulator. Further research by Lewis et al. and Giannotti et al. use a 3D Systems’ ‘Lap Mentor’. Despite their small sample sizes, these papers validate the use of the Lap Mentor in the training and assessment of bariatric surgeons. No other research exploring the application of VR simulators outside bariatric surgery was identified. The use of 3D printing within simulators was not described by any studies.

3D reconstruction techniques have been used to visualise both normal and pathological anatomy for surgeons. Kavic et al. used CT images to create 3D virtual models of hiatus hernias, hoping to aid the understanding of hiatus hernia classifications and perhaps advance existing classification systems. Using publicly available cadaveric datasets, Wu et al. and Shin et al. generated virtual 3D models of the thorax and gastro-intestinal tract, including stomach and oesophagus. Using similar methods, Kwon et al. created virtual endoscopic and laparoscopic simulations of stomach wall anatomy. These papers conclude that virtual 3D reconstructions give junior trainees a deeper understanding of complex anatomical relationships. However, these insights were not supported by quantitative comparisons to traditional educational tools.

In summary, VR simulators have been shown to be an effective tool for the training and certification of bariatric surgeons. However, the application of these simulators to other oesophagogastric surgical procedures is yet to be validated. Virtual 3D reconstructions of oesophagogastric anatomy and pathology have been created, but there is no evidence for their superiority over traditional training methods. Furthermore, physical 3D printed models have yet to be used in the education and training of oesophagogastric surgeons.

### Patient engagement and education

The scoping review did not identify any literature that focussed on the application of 3D modelling to patient engagement and education in oesophagogastric surgery. In the conclusion of the case series by Dickenson et al., patient engagement is highlighted as a significant advantage to 3D printed models. Patient engagement is a fundamental application of 3D modelling and disappointingly appears under-researched in oesophagogastric surgery.

## Discussion

This scoping review identified and critically reviewed 56 papers in respect to the use of 3D reconstruction and its current and future role within oesophagogastric surgery. The review demonstrates 3D virtual modelling can be used for radiological diagnosis and operative guidance, including vascular reconstruction, in oesophagogastric surgery. The predominant focus on vascular reconstructions likely reflects the relative ease of modelling contrast enhanced vascular structures in comparison to hollow viscera.

Although the subjective benefit of 3D modelling for operative planning is described, few studies demonstrate improved surgical outcomes. For 3D reconstruction to become routine in oesophagogastric surgery, well powered RCTs are essential. Researchers should consider emulating encouraging work in other surgical specialties, such as urology, where a recent RCT demonstrated 3D modelling reduces operative time, blood loss and length of hospital stay [85].

Critics of 3D reconstruction suggest the high cost and time demands of this technique will prevent its wider adoption. Only one study in this review quantified these variables. However, cost-analysis in other surgical specialties has shown the resource-intensive requirements of 3D modelling are matched by downstream savings [17]. Researchers within oesophagogastric surgery should therefore consider a formal cost-analysis to assess the impact of 3D reconstruction on healthcare provision.

Although the segmentation process of 3D reconstruction is highly labour intensive, machine learning methodologies have demonstrated fully automated segmentation is feasible for abdominal viscera and vasculature [86–88]. Considering the possible healthcare savings identified in other areas, the development of automated segmentation methods and falling technology costs, it would be prudent to quantify the potential benefits of 3D reconstruction in oesophagogastric surgical practice as the technology becomes easier to adopt.

As surgical training evolves, it moves further from the traditional apprenticeship model [89]. Modern surgical trainees lack the experiential opportunities of their predecessors, and it is hoped surgical simulators could replace this. Training simulators using either virtual or 3D printed reconstructions offer a cheaper and more ethically acceptable alternative to cadaveric or animal-based simulators [90]. With the successful validation of VR simulators for bariatric surgery, it would be sensible to assess their role in bariatric surgical training programmes. This scoping review shows the need to validate VR simulators for oesophagogastric procedures beyond bariatric surgery. Furthermore, there is currently no published research exploring the use of 3D printed surgical simulators in oesophagogastric training.

3D reconstructions, either virtual of physical, can provide significant anatomical educational benefits to medical students and surgeons alike. Innovative applications of 3D reconstructions have been shown to provide an advantage over conventional educational practices in several surgical specialities [6, 91–93]. In comparison, this technique appears underutilised within oesophagogastric surgery. This review only identified 4 studies using 3D reconstruction for anatomical education, none of which demonstrated a quantifiable advantage. However, 3D modelling might help junior surgeons to understand the intricacies of complex oesophagogastric procedures and this should be evaluated in future work.

3D reconstruction offers substantial value to patient engagement and education. Disappointingly this is underutilised in oesophagogastric surgery. Many other surgical specialities have explored the application of 3D reconstruction for patient engagement [12, 94–99]. Those studies consistently show 3D reconstructions, especially 3D printed models, improve patients’ understanding of their pathology and proposed treatment. It is likely that generic or patient-specific 3D models would be useful tools for securing informed consent for oesophagogastric surgery. Future research should evaluate the application of 3D reconstructions for oesophagogastric surgical patients with particular attention paid to the importance of generic versus patient-specific models.

From a technological perspective, software and reconstruction methodologies were poorly described in the papers reviewed. This is important. Without clear descriptions of the technology and techniques used, a framework for future researchers cannot be constructed or recommended. The predominance of volume rendering over surface rendering likely reflects the comparative simplicity volume rendering provides over surface rendering [45, 100]. A future review detailing the technology and reconstruction techniques best suited to oesophagogastric surgery would be highly valuable to novices in this field.

Only 5% of the studies in this scoping review used 3D printed models and only in the context of operating planning. There are no direct comparisons between virtual and physical 3D models, therefore no conclusions can be drawn about superiority between the two techniques in oesophagogastric surgery. However, it is likely that neither technique is superior overall, instead the choice is dependent on the theme researched. For example, for intra-operative guidance, an augmented reality model is likely to provide greater value than a 3D printed model to the operating and scrubbed surgeon. In contrast, for pre-operative guidance, there is early evidence to suggest 3D printed models provide greater benefit for surgical planning across a range of specialties [101, 102]. Similarly, some work suggests patients gain a greater understanding when shown 3D printed models rather than virtual alternatives [98]. In simulation training, there are considerable benefits and challenges to both virtual and 3D printed simulators [103]. The decision between the two is likely to depend on the skill being taught and the training centre itself. Whilst the findings of this review suggest 3D printing is underutilized in oesophagogastric surgery, both forms of 3D modelling are valuable tools to researchers.

Unfortunately, this review identified little high-quality research into 3D reconstruction. Compared to other surgical specialties (Table 2 [101, 104–166]), oesophagogastric surgery has fallen far behind. Without a concerted effort to correct this, the specialty might miss the opportunity to take advantage of the multi-faceted applications of 3D modelling.

**Table 2:** Comparison of published randomised control trials and systematic reviews in 3D reconstruction between surgical specialties

*Specialty*	Number of randomised control trials in 3D reconstruction*	Number of systematic reviews in 3D reconstruction*
*Orthopaedic surgery*	37	7
*Hepatobiliary surgery*	7	4
*Urology*	5	4
*Oesophagogastric surgery*	0	0

^*^Studies identified through PubMed search between 2010 and 2020

### Limitations

This scoping review has several acknowledged limitations. Most of the included studies originated from Asia. As this paper was limited to the English language, publications not translated into English may have been excluded, introducing a selection bias. Furthermore, as this scoping review focussed on the application of 3D reconstruction in relation to surgical settings, innovations within other medical fields applicable to surgery may also have been excluded. In the construction of the protocol, it was determined that studies should not be excluded based on a methodological quality analysis. Whilst this allowed a breadth of studies to be included for critical analysis, it may allow studies of poor quality to be overrepresented.

### Conclusions

This is the first review, to the authors’ knowledge, summarising the current and future roles of 3D reconstruction within oesophagogastric surgery. Clearly, 3D modelling is in its infancy compared to other surgical specialties. There is early promising evidence suggesting 3D reconstruction could offer significant benefit to oesophagogastric surgery. However, without further high-quality research in this field, the specialty may be left behind. This would be detrimental to all parties including patients, trainees and established oesophagogastric surgeons.

## Supplementary Information

Below is the link to the electronic supplementary material.

Supplementary file1 (DOCX 14 KB)

Supplementary file2 (DOCX 13 KB)

Supplementary file3 (DOCX 28 KB)

## References

[1] Alberti C (1980). Three-dimensional CT and structure models. Br J Radiol.

[2] Kim N, Lee S, Gwon E, Seo JB (2020). The value in 3D printing. Med Radiol.

[3] Landini L, Positano V, Santarelli MF, Neri E, Caramella D, Bartolozzi C (2008). 3D medical image processing. Image Processing in radiology: current applications.

[4] Bernardo A (2017). Virtual reality and simulation in neurosurgical training. World Neurosurg.

[5] Moglia A, Di Franco G, Morelli L (2019). Use of 3D models for planning, simulation, and training in vascular surgery. Updat Surg.

[6] Sahnan K, Adegbola SO, Tozer PJ, Gupta A, Baldwin-Cleland R, Yassin N (2018). Improving the understanding of perianal crohn fistula through 3D modeling. Ann Surg.

[7] Shen S, Wang P, Li X, Han X, Tan H (2020). Pre-operative simulation using a three-dimensional printing model for surgical treatment of old and complex tibial plateau fractures. Sci Rep.

[8] Kneebone RL (2016). Simulation reframed. Adv Simul.

[9] Lemole GM, Banerjee PP, Luciano C, Neckrysh S, Charbel FT (2007). Virtual reality in neurosurgical education: part-task ventriculostomy simulation with dynamic visual and haptic feedback. Neurosurgery.

[10] Zhuang YD, Zhou MC, Liu SC, Wu JF, Wang R, Chen CM (2019). Effectiveness of personalized 3D printed models for patient education in degenerative lumbar disease. Patient Educ Couns.

[11] Pandrangi VC, Gaston B, Appelbaum NP, Albuquerque FC, Levy MM, Larson RA (2019). The application of virtual reality in patient education. Ann Vasc Surg.

[12] van de Belt TH, Nijmeijer H, Grim D, Engelen L, Vreeken R, van Gelder M (2018). Patient-specific actual-size three-dimensional printed models for patient education in glioma treatment: first experiences. World Neurosurg.

[13] Shuhaiber JH (2004). Augmented reality in surgery. Arch Surg.

[14] Barlow J (2016) Managing Innovation in Healthcare. World Scientific, Europe. p: 444

[15] Papazarkadas X, Spartalis E, Patsouras D, Ioannidis A, Schizas D, Georgiou K (2019). The role of 3D printing in colorectal surgery: current evidence and future perspectives. In vivo (Athens, Greece).

[16] Martelli N, Serrano C, van den Brink H, Pineau J, Prognon P, Borget I (2016). Advantages and disadvantages of 3-dimensional printing in surgery: systematic review. Surgery.

[17] Ballard DH, Mills P, Duszak R, Weisman JA, Rybicki FJ, Woodard PK (2020). Medical 3D printing cost-savings in orthopedic and maxillofacial surgery: cost analysis of operating room time saved with 3D printed anatomic models and surgical guides. Acad Radiol.

[18] Sucharew H, Macaluso M (2019). Progress notes: methods for research evidence synthesis: the scoping review approach. J Hosp Med.

[19] Munn Z, Peters MDJ, Stern C, Tufanaru C, McArthur A, Aromataris E (2018). Systematic review or scoping review? Guidance for authors when choosing between a systematic or scoping review approach. BMC Med Res Methodol.

[20] Arksey H, O'Malley L (2005). Scoping studies: towards a methodological framework. Int J Soc Res Methodol.

[21] Levac D, Colquhoun H, O'Brien KK (2010). Scoping studies: advancing the methodology. Implement Sci.

[22] Peters MDJGC, McInerney P, Munn Z, Tricco AC, Khalil H, Aromataris E, Munn Z (2020). Chapter 11: Scoping Reviews. JBI Manual for Evidence Synthesis.

[23] Robb HD, Scrimgeour G, Boshier PR, Balyasnikova S, Brown G, Bello F (2021). Current and possible future role of 3D modelling within oesophagogastric surgery: a scoping review protocol. BMJ Open.

[24] Peters MDJ, Godfrey CM, Khalil H, McInerney P, Parker D, Soares CB (2015). Guidance for conducting systematic scoping reviews. Int J Evid Based Healthc.

[25] Tricco AC, Lillie E, Zarin W, O'Brien KK, Colquhoun H, Levac D (2018). PRISMA extension for scoping reviews (PRISMA-ScR): checklist and explanation. Ann Intern Med.

[26] Innovation VH. Covidence systematic review software. Melbourne: Veritas Health Innovation; 2020.

[27] Moher D, Liberati A, Tetzlaff J, Altman DG (2009). Preferred reporting items for systematic reviews and meta-analyses: the PRISMA statement. BMJ.

[28] Sankaranarayanan G, Adair JD, Halic T, Gromski MA, Lu Z, Ahn W (2011). Validation of a novel laparoscopic adjustable gastric band simulator. Surg Endosc.

[29] Lewis TM, Aggarwal R, Kwasnicki RM, Rajaretnam N, Moorthy K, Ahmed A (2012). Can virtual reality simulation be used for advanced bariatric surgical training?. Surgery.

[30] Giannotti D, Patrizi G, Casella G, Di Rocco G, Marchetti M, Frezzotti F (2014). Can virtual reality simulators be a certification tool for bariatric surgeons?. Surg Endosc.

[31] Choi C, Kim J, Han H, Ahn B, Kim J (2009). Graphic and haptic modelling of the oesophagus for VR-based medical simulation. Int J Med Robot.

[32] Kavic SM, Segan RD, George IM, Turner PL, Roth JS, Park A (2006). Classification of hiatal hernias using dynamic three-dimensional reconstruction. Surg Innov.

[33] Shin DS, Park JS, Lee S-B, Lee S-H, Chung J, Chung MS (2009). Surface model of the gastrointestinal tract constructed from the Visible Korean. Clin Anat.

[34] Kwon K, Shin DS, Shin BS, Park HS, Lee S, Jang HG (2015). Virtual endoscopic and laparoscopic exploration of stomach wall based on a cadaver's sectioned images. J Korean Med Sci.

[35] Wu Y, Luo N, Tan L, Fang B, Li Y, Xie B (2013). Three-dimensional reconstruction of thoracic structures: based on Chinese Visible Human. Comput Math Methods Med.

[36] Usui S, Hiranuma S, Ichikawa T, Maeda M, Kudo S-E, Iwai T (2005). Preoperative imaging of surrounding arteries by three-dimensional CT: is it useful for laparoscopic gastrectomy?. Surg Laparosc Endosc Percutaneous Tech.

[37] Matsuo K, Inoue M, Shirai Y, Kataoka T, Kagota S, Taniguchi K (2018). Giant GIST of the stomach: a successful case of safe resection with preoperative simulation using three-dimensional CT angiography: Case report. Medicine.

[38] Lee S-W, Shinohara H, Matsuki M, Okuda J, Nomura E, Mabuchi H (2003). Preoperative simulation of vascular anatomy by three-dimensional computed tomography imaging in laparoscopic gastric cancer surgery. J Am Coll Surg.

[39] Matsuki M, Kani H, Tatsugami F, Yoshikawa S, Narabayashi I, Lee S-W (2004). Preoperative assessment of vascular anatomy around the stomach by 3D imaging using MDCT before laparoscopy-assisted gastrectomy. AJR Am J Roentgenol.

[40] Matsuki M, Tanikake M, Kani H, Tatsugami F, Kanazawa S, Kanamoto T (2006). Dual-phase 3D CT angiography during a single breath-hold using 16-MDCT: assessment of vascular anatomy before laparoscopic gastrectomy. AJR Am J Roentgenol.

[41] Zheng Y, Zhao X-W, Zhang H-L, Wang Z-H, Wang Y (2018). An acquired transposition of the aortic arch secondary to large esophageal cancer misdiagnosed as a right-side aortic arch. J Thorac Dis.

[42] Li X, Chu J, Sun C, Pui MH, Huang S, Feng S (2013). Sixty-four-slice computed tomography angiography of perigastric veins with image fusion. J Comput Assist Tomogr.

[43] Huang C-M, Wang J-B, Wang Y, Zheng C-H, Li P, Xie J-W (2014). Left gastric vein on the dorsal side of the splenic artery: a rare anatomic variant revealed during gastric surgery. Surg Radiol Anat.

[44] Wang P, Zhang CZ, Wang GB, Li YY, Jiang XY, Fang FJ (2018). Evaluation of computed tomography vascular reconstruction for the localization diagnosis of perigastric mass. Medicine (United States)..

[45] Zhu C, Kong S-H, Kim T-H, Park S-H, Ang RRG, Diana M (2018). The anatomical configuration of the splenic artery influences suprapancreatic lymph node dissection in laparoscopic gastrectomy: analysis using a 3D volume rendering program. Surg Endosc.

[46] Wu D, Zhao L, Liu Y, Wang J, Hu W, Feng X (2017). The superiority of 256-slice spiral computed tomography angiography for preoperative evaluation of surrounding arteries in patients with gastric cancer. Onco Targets Ther.

[47] Sunagawa H, Tokunagawa M, Kaito A, Kinoshita T (2018). How to apply three-dimensional computed tomography simulation for laparoscopic lymphadenectomy around the splenic hilum for gastric cancer. Surg Endosc.

[48] Peng J, Xiang Z-J, Ren G-G, Xiao B, Zhu J, Rong H (2018). Successful resection of esophageal carcinoma with a double aortic arch. Ann Thorac Surg.

[49] Wang J-B, Huang C-M, Zheng C-H, Li P, Xie J-W, Lin J-X (2014). Role of 3DCT in laparoscopic total gastrectomy with spleen-preserving splenic lymph node dissection. World J Gastroenterol.

[50] Song YN, Qi Y, Zhang CY, Sheng YL, Wu K, Yang Y (2019). Case report a new model for reducing the risk of surgery for oesophageal cancer with right-side aortic arch-3d reconstruction. Int J Clin Exp Med.

[51] Kinoshita T, Shibasaki H, Enomoto N, Sahara Y, Sunagawa H, Nishida T (2016). Laparoscopic splenic hilar lymph node dissection for proximal gastric cancer using integrated three-dimensional anatomic simulation software. Surg Endosc.

[52] Han NY, Park BJ, Park S-S, Sung DJ, Kim MJ, Cho SB (2014). Modified fusion imaging combining CT gastrography and CT angiography: an initial experience of preoperative mapping prior to laparoscopic exogastric wedge resection of small (<3 cm) gastric submucosal lesions. Abdom Imaging.

[53] Chang J-M, Yoo YS, Kim D-W (2011). Application of three-dimensional reconstruction in esophageal foreign bodies. Korean J Thorac Cardiovasc Surg.

[54] Bosque Lopez MJ, Llompart Rigo A, de-Miguel-Sebastian P, (2010). A foreign body in the esophagus. Rev Esp Enferm Dig.

[55] Wong YM, Makmur A, Lau LC, Ting E (2018). Temporal evolution of intramural esophageal dissection with 3D reconstruction and cinematic virtual fly-through. J Radiol Case Rep.

[56] Chen D, Tian S, Hu Z, Wu J (2020). Cardia laxity under retroflexed endoscopy is a reflection of esophageal hiatus enlargement. Gastroenterol Res Practice.

[57] Adachi Y, Akino K, Mita H, Kikuchi T, Endo T (2013). Computed tomography just after endoscopy as the preoperative examination for safe percutaneous endoscopic gastrostomy. Digestion.

[58] Takanami K, Ichikawa H, Fukuda H, Takahashi S (2012). Three-dimensional lymphoscintigraphy using spect/ct and 123i-bmipp for the preoperative detection of anatomical anomalies of the thoracic duct. Clin Nucl Med.

[59] Kato T, Takase K, Ichikawa H, Satomi S, Takahashi S (2011). Thoracic duct visualization: Combined use of multidetector-row computed tomography and magnetic resonance imaging. J Comput Assist Tomogr.

[60] Disse E, Pasquer A, Pelascini E, Valette P-J, Betry C, Laville M (2017). Dilatation of sleeve gastrectomy: myth or reality?. Obes Surg.

[61] Santander C, Awad W, Garay A, Martinez C (2015). 3D-msct gastric pouch volumetry in banded gastric bypass: Preliminary clinical results. Obes Surg.

[62] Huh J, Lee I-S, Kim KW, Park J, Kim AY, Lee JS (2016). CT gastrography for volumetric measurement of remnant stomach after distal gastrectomy: a feasibility study. Abdom Radiol.

[63] Cai H, Wang R, Li Y, Yang X, Cui Y (2018). Role of 3D reconstruction in the evaluation of patients with lower segment oesophageal cancer. J Thorac Dis.

[64] Bean MJ, Horton KM, Fishman EK (2005). Detection and diagnosis of gastric carcinoma with multidetector and 3D computed tomography. Appl Radiol.

[65] Onbaş O, Eroglu A, Kantarci M, Polat P, Alper F, Karaoglanoglu N (2006). Preoperative staging of esophageal carcinoma with multidetector CT and virtual endoscopy. Eur J Radiol.

[66] Kim JH, Eun HW, Hong SS, Auh YH (2006). Early gastric cancer: virtual gastroscopy. Abdom Imaging.

[67] Ahn HS, Kim SH, Kodera Y, Yang H-K (2013). Gastric cancer staging with radiologic imaging modalities and UICC staging system. Dig Surg.

[68] Choi J-I, Joo I, Lee JM (2014). State-of-the-art preoperative staging of gastric cancer by MDCT and magnetic resonance imaging. World J Gastroenterol.

[69] Lee IJ, Lee JM, Kim SH, Shin C-I, Lee JY, Kim SH (2010). Diagnostic performance of 64-channel multidetector CT in the evaluation of gastric cancer: differentiation of mucosal cancer (T1a) from submucosal involvement (T1b and T2). Radiology.

[70] Mamede M, El Fakhri G, Abreu-e-Lima P, Gandler W, Nose V, Gerbaudo VH (2007). Pre-operative estimation of esophageal tumor metabolic length in FDG-PET images with surgical pathology confirmation. Ann Nucl Med.

[71] Park HS, Lee JM, Kim SH, Lee JY, Yang H-K, Han JK (2010). Three-dimensional MDCT for preoperative local staging of gastric cancer using gas and water distention methods: a retrospective cohort study. AJR Am J Roentgenol.

[72] Chen B-B, Liang P-C, Liu K-L, Hsiao J-K, Huang J-C, Wong J-M (2007). Preoperative diagnosis of gastric tumors by three-dimensional multidetector row ct and double contrast barium meal study: correlation with surgical and histologic results. J Formosan Med Association.

[73] Kim JH, Park SH, Hong HS, Auh YH (2005). CT gastrography. Abdom Imaging.

[74] Alfieri R, Pintacuda G, Cagol M, Occhipinti T, Capraro I, Scarpa M (2015). Oesophageal cancer: assessment of tumour response to chemoradiotherapy with tridimensional CT. Radiol Med (Torino).

[75] Lee MW, Kim SH, Kim YJ, Lee JM, Lee JY, Park E-A (2008). Gastrointestinal stromal tumor of the stomach: preliminary results of preoperative evaluation with CT gastrography. Abdom Imaging.

[76] Kim AY, Kim HJ, Ha HK (2005). Gastric cancer by multidetector row CT: Preoperative staging. Abdom Imaging.

[77] Singh AK, Hiroyuki Y, Sahani DV (2009). Advanced postprocessing and the emerging role of computer-aided detection. Radiol Clin North Am.

[78] Duan S-Y, Zhang D-T, Lin Q-C, Wu Y-H (2006). Clinical value of CT three-dimensional imaging in diagnosing gastrointestinal tract diseases. World J Gastroenterol.

[79] Marano L, Ricci A, Savelli V, Verre L, Di Renzo L, Biccari E (2019). From digital world to real life: a robotic approach to the esophagogastric junction with a 3D printed model. BMC Surg.

[80] Ye L, Yang D, Huang Y, Liao K, Yuan X, Hu B (2020). 3D-printed model in the guidance of tumor resection: a novel concept for resecting a large submucosal tumor in the mid-esophagus. Endoscopy.

[81] Dickinson KJ, Cassivi SD, Reinersman JM, Matsumoto JS, Fletcher JG, Morris J (2015). Individualizing management of complex esophageal pathology using 3D printed anatomic models. Gastroenterology.

[82] Sato Y, Sugimoto M, Tanaka Y, Suetsugu T, Imai T, Hatanaka Y (2020). Holographic image-guided thoracoscopic surgery: possibility of usefulness for esophageal cancer patients with abnormal artery. Esophagus.

[83] Kim YM, Baek S-E, Lim JS, Hyung WJ (2013). Clinical application of image-enhanced minimally invasive robotic surgery for gastric cancer: a prospective observational study. J Gastrointest Surg.

[84] Burns PB, Rohrich RJ, Chung KC (2011). The levels of evidence and their role in evidence-based medicine. Plast Reconstr Surg.

[85] Shirk JD, Thiel DD, Wallen EM, Linehan JM, White WM, Badani KK (2019). Effect of 3-dimensional virtual reality models for surgical planning of robotic-assisted partial nephrectomy on surgical outcomes: a randomized clinical trial. JAMA Netw Open.

[86] Oda M, Roth HR, Kitasaka T, Misawa K, Fujiwara M, Mori K (2019). Abdominal artery segmentation method from CT volumes using fully convolutional neural network. Int J Comput Assist Radiol Surg.

[87] Roth HR, Oda H, Zhou X, Shimizu N, Yang Y, Hayashi Y (2018). An application of cascaded 3D fully convolutional networks for medical image segmentation. Comput Med Imaging Graph.

[88] Kitasaka T, Kagajo M, Nimura Y, Hayashi Y, Oda M, Misawa K (2017). Automatic anatomical labeling of arteries and veins using conditional random fields. Int J Comput Assist Radiol Surg.

[89] W H. THE TRAINING OF THE SURGEON. Journal of the American Medical Association. 1904;XLIII(21):1553–4.

[90] Przedlacka A, Korzeniowski P, Tekkis P, Bello F, Kontovounisios C, Atallah S (2021). 3D simulation and modeling for surgeon education and patient engagement. Digital Surgery.

[91] Wellens LM, Meulstee J, van de Ven CP, Terwisscha van Scheltinga CEJ, Littooij AS, van den Heuvel-Eibrink MM (2019). Comparison of 3-dimensional and augmented reality kidney models with conventional imaging data in the preoperative assessment of children with wilms tumors. JAMA Netw Open.

[92] Crossingham JL, Jenkinson J, Woolridge N, Gallinger S, Tait GA, Moulton CA (2009). Interpreting three-dimensional structures from two-dimensional images: a web-based interactive 3D teaching model of surgical liver anatomy. HPB (Oxford).

[93] Wada Y, Nishi M, Yoshikawa K, Higashijima J, Miyatani T, Tokunaga T (2020). Usefulness of virtual three-dimensional image analysis in inguinal hernia as an educational tool. Surg Endosc.

[94] Khural M, Gullipalli R, Dubrowski A (2020). Evaluating the use of a generic three-dimensionally (3D) printed abdominal aortic aneurysm model as an adjunct patient education tool. Cureus..

[95] Yang T, Tan T, Yang J, Pan J, Hu C, Li J (2018). The impact of using three-dimensional printed liver models for patient education. J Int Med Res.

[96] Sezer S, Piai V, Kessels RPC, Ter Laan M. Information recall in pre-operative consultation for glioma surgery using actual size three-dimensional models. J Clin Med. 2020;9(11). 10.3390/jcm9113660 PMC7698093 33203047

[97] Sander IM, Liepert TT, Doney EL, Leevy WM, Liepert DR. Patient education for endoscopic sinus surgery: preliminary experience using 3D-printed clinical imaging data. J Funct Biomater. 2017;8(2). 10.3390/jfb8020013 PMC5491994 28387702

[98] Wake N, Rosenkrantz AB, Huang R, Park KU, Wysock JS, Taneja SS (2019). Patient-specific 3D printed and augmented reality kidney and prostate cancer models: impact on patient education. 3D Print Med.

[99] Bernhard JC, Isotani S, Matsugasumi T, Duddalwar V, Hung AJ, Suer E (2016). Personalized 3D printed model of kidney and tumor anatomy: a useful tool for patient education. World J Urol.

[100] Fishman EK, Ney DR, Heath DG, Corl FM, Horton KM, Johnson PT (2006). Volume rendering versus maximum intensity projection in CT angiography: what works best, when, and why. Radiographics.

[101] Zheng YX, Yu DF, Zhao JG, Wu YL, Zheng B (2016). 3D printout models vs 3D-rendered images: which is better for preoperative planning?. J Surg Educ.

[102] Pugliese L, Marconi S, Negrello E, Mauri V, Peri A, Gallo V (2018). The clinical use of 3D printing in surgery. Updat Surg.

[103] Michaels R, Witsberger CA, Powell AR, Koka K, Cohen K, Nourmohammadi Z (2021). 3D printing in surgical simulation emphasized importance in the COVID-19 pandemic era. J 3D Print Med.

[104] Abdel MP, Parratte S, Blanc G, Ollivier M, Pomero V, Viehweger E (2014). No benefit of patient-specific instrumentation in TKA on functional and gait outcomes: a randomized clinical trial. Clin Orthop Relat Res.

[105] Ali S, Sirota E, Ali H, Bezrukov E, Okhunov Z, Bukatov M (2020). Three-dimensionally printed non-biological simulator for percutaneous nephrolithotomy training. Scand J Urol.

[106] Baier C, Springorum HR, Götz J, Schaumburger J, Lüring C, Grifka J (2013). Comparing navigation-based in vivo knee kinematics pre- and postoperatively between a cruciate-retaining and a cruciate-substituting implant. Int Orthop.

[107] Bayraktar V, Weber M, von Kunow F, Zeman F, Craiovan B, Renkawitz T (2017). Accuracy of measuring acetabular cup position after total hip arthroplasty: comparison between a radiographic planning software and three-dimensional computed tomography. Int Orthop.

[108] Beerekamp MS, Ubbink DT, Maas M, Luitse JS, Kloen P, Blokhuis TJ (2011). Fracture surgery of the extremities with the intra-operative use of 3D-RX: a randomized multicenter trial (EF3X-trial). BMC Musculoskelet Disord.

[109] Bertolo R, Hung A, Porpiglia F, Bove P, Schleicher M, Dasgupta P (2020). Systematic review of augmented reality in urological interventions: the evidences of an impact on surgical outcomes are yet to come. World J Urol.

[110] Brenneis M, Braun S, van Drongelen S, Fey B, Tarhan T, Stief F (2021). Accuracy of preoperative templating in total hip arthroplasty with special focus on stem morphology: a randomized comparison between common digital and three-dimensional planning using biplanar radiographs. J Arthroplasty.

[111] Briem D, Ruecker AH, Neumann J, Gebauer M, Kendoff D, Gehrke T (2011). 3D fluoroscopic navigated reaming of the glenoid for total shoulder arthroplasty (TSA). Comput Aided Surg.

[112] Chen C, Cai L, Zhang C, Wang J, Guo X, Zhou Y (2018). Treatment of die-punch fractures with 3D printing technology. J Invest Surg.

[113] Chen C, Cai L, Zheng W, Wang J, Guo X, Chen H (2019). The efficacy of using 3D printing models in the treatment of fractures: a randomised clinical trial. BMC Musculoskelet Disord.

[114] Cicione A, Autorino R, Laguna MP, De Sio M, Micali S, Turna B (2015). Three-dimensional technology facilitates surgical performance of novice laparoscopy surgeons: a quantitative assessment on a porcine kidney model. Urology.

[115] de Muinck Keizer RJO, Lechner KM, Mulders MAM, Schep NWL, Eygendaal D, Goslings JC (2017). Three-dimensional virtual planning of corrective osteotomies of distal radius malunions: a systematic review and meta-analysis. Strategies Trauma Limb Reconstr.

[116] De Vloo R, Pellikaan P, Dhollander A, Vander SJ (2017). Three-dimensional analysis of accuracy of component positioning in total knee arthroplasty with patient specific and conventional instruments: A randomized controlled trial. Knee.

[117] Fang CH, Kong D, Wang X, Wang H, Xiang N, Fan Y (2014). Three-dimensional reconstruction of the peripancreatic vascular system based on computed tomographic angiography images and its clinical application in the surgical management of pancreatic tumors. Pancreas.

[118] Hallet J, Gayet B, Tsung A, Wakabayashi G, Pessaux P (2015). Systematic review of the use of pre-operative simulation and navigation for hepatectomy: current status and future perspectives. J Hepatobiliary Pancreat Sci.

[119] Hasan S, van Hamersveld KT, Marang-van de Mheen PJ, Kaptein BL, Nelissen R, Toksvig-Larsen S (2020). Migration of a novel 3D-printed cementless versus a cemented total knee arthroplasty two-year results of a randomized controlled trial using radiostereometric analysis. Bone Joint J..

[120] Huang JH, Liao H, Tan XY, Xing WR, Zhou Q, Zheng YS (2020). Surgical treatment for both-column acetabular fractures using pre-operative virtual simulation and three-dimensional printing techniques. Chin Med J (Engl).

[121] Iannotti JP, Walker K, Rodriguez E, Patterson TE, Jun BJ, Ricchetti ET (2019). Accuracy of 3-dimensional planning, implant templating, and patient-specific instrumentation in anatomic total shoulder arthroplasty. J Bone Joint Surg Am.

[122] Jiang M, Chen G, Coles-Black J, Chuen J, Hardidge A (2020). Three-dimensional printing in orthopaedic preoperative planning improves intraoperative metrics: a systematic review. ANZ J Surg.

[123] Jin H, Xu R, Wang J (2019). The effects of short-term wearing of customized 3D printed single-sided lateral wedge insoles on lower limbs in healthy males: a randomized controlled trial. Med Sci Monit.

[124] Ke S, Ran T, He Y, Lv M, Song X, Zhou Y (2020). Does patient-specific instrumentation increase the risk of notching in the anterior femoral cortex in total knee arthroplasty? A comparative prospective trial. Int Orthop.

[125] Kim SJ, Kim SJ, Cha YH, Lee KH, Kwon JY (2018). Effect of personalized wrist orthosis for wrist pain with three-dimensional scanning and printing technique: A preliminary, randomized, controlled, open-label study. Prosthet Orthot Int.

[126] Kong L, Yang G, Yu J, Zhou Y, Li S, Zheng Q (2020). Surgical treatment of intra-articular distal radius fractures with the assistance of three-dimensional printing technique. Medicine (Baltimore).

[127] Kong X, Nie L, Zhang H, Wang Z, Ye Q, Tang L (2016). Do 3D printing models improve anatomical teaching about hepatic segments to medical students? A randomized controlled study. World J Surg.

[128] Kong X, Nie L, Zhang H, Wang Z, Ye Q, Tang L (2016). Do Three-dimensional visualization and three-dimensional printing improve hepatic segment anatomy teaching? a randomized controlled study. J Surg Educ.

[129] Kowalewski KF, Garrow CR, Proctor T, Preukschas AA, Friedrich M, Müller PC (2018). LapTrain: multi-modality training curriculum for laparoscopic cholecystectomy-results of a randomized controlled trial. Surg Endosc.

[130] Lal H, Patralekh MK (2018). 3D printing and its applications in orthopaedic trauma: A technological marvel. J Clin Orthop Trauma.

[131] Laverdière C, Corban J, Khoury J, Ge SM, Schupbach J, Harvey EJ (2019). Augmented reality in orthopaedics: a systematic review and a window on future possibilities. Bone Joint J.

[132] Li A, Tang R, Rong Z, Zeng J, Xiang C, Yu L (2018). The use of three-dimensional printing model in the training of choledochoscopy techniques. World J Surg.

[133] Lin C, Gao J, Zheng H, Zhao J, Yang H, Lin G (2020). Three-dimensional visualization technology used in pancreatic surgery: a valuable tool for surgical trainees. J Gastrointest Surg.

[134] Lv H, Zhang L, Yang F, Li M, Yin P, Su X (2015). A novel 3D-printed device for localization and extraction of trabeculae from human femoral heads: a comparison with traditional visual extraction. Osteoporos Int.

[135] Maini L, Sharma A, Jha S, Sharma A, Tiwari A (2018). Three-dimensional printing and patient-specific pre-contoured plate: future of acetabulum fracture fixation?. Eur J Trauma Emerg Surg.

[136] Maini L, Verma T, Sharma A, Sharma A, Mishra A, Jha S (2018). Evaluation of accuracy of virtual surgical planning for patient-specific pre-contoured plate in acetabular fracture fixation. Arch Orthop Trauma Surg.

[137] Manning TG, O'Brien JS, Christidis D, Perera M, Coles-Black J, Chuen J (2018). Three dimensional models in uro-oncology: a future built with additive fabrication. World J Urol.

[138] Maus U, Marques CJ, Scheunemann D, Lampe F, Lazovic D, Hommel H (2018). No improvement in reducing outliers in coronal axis alignment with patient-specific instrumentation. Knee Surg Sports Traumatol Arthrosc.

[139] Ollivier M, Tribot-Laspiere Q, Amzallag J, Boisrenoult P, Pujol N, Beaufils P (2016). Abnormal rate of intraoperative and postoperative implant positioning outliers using "MRI-based patient-specific" compared to "computer assisted" instrumentation in total knee replacement. Knee Surg Sports Traumatol Arthrosc.

[140] Perica ER, Sun Z (2018). A systematic review of three-dimensional printing in liver disease. J Digit Imaging.

[141] Rai A, Scovell JM, Xu A, Balasubramanian A, Siller R, Kohn T (2018). Patient-specific virtual simulation-a state of the art approach to teach renal tumor localization. Urology.

[142] Renkawitz T, Haimerl M, Dohmen L, Gneiting S, Wegner M, Ehret N (2011). Minimally invasive computer-navigated total hip arthroplasty, following the concept of femur first and combined anteversion: design of a blinded randomized controlled trial. BMC Musculoskelet Disord.

[143] Sariali E, Boukhelifa N, Catonne Y, Pascal MH (2016). Comparison of three-dimensional planning-assisted and conventional acetabular cup positioning in total hip arthroplasty: a randomized controlled trial. J Bone Joint Surg Am.

[144] Sariali E, Kajetanek C, Catonné Y (2019). Comparison of custom cutting guides based on three-dimensional computerized CT-scan planning and a conventional ancillary system based on two-dimensional planning in total knee arthroplasty: a randomized controlled trial. Int Orthop.

[145] Sariali E, Mauprivez R, Khiami F, Pascal-Mousselard H, Catonné Y (2012). Accuracy of the preoperative planning for cementless total hip arthroplasty. A randomised comparison between three-dimensional computerised planning and conventional templating. Orthop Traumatol Surg Res.

[146] Schout BM, Ananias HJ, Bemelmans BL, d'Ancona FC, Muijtjens AM, Dolmans VE (2010). Transfer of cysto-urethroscopy skills from a virtual-reality simulator to the operating room: a randomized controlled trial. BJU Int.

[147] Shirk JD, Thiel DD, Wallen EM, Linehan JM, White WM, Badani KK (2019). Effect of 3-dimensional virtual reality models for surgical planning of robotic-assisted partial nephrectomy on surgical outcomes: a randomized clinical trial. JAMA Netw Open.

[148] Shuang F, Hu W, Shao Y, Li H, Zou H (2016). Treatment of intercondylar humeral fractures with 3D-printed osteosynthesis plates. Medicine (Baltimore).

[149] Small T, Krebs V, Molloy R, Bryan J, Klika AK, Barsoum WK (2014). Comparison of acetabular shell position using patient specific instruments vs. standard surgical instruments: a randomized clinical trial. J Arthroplasty.

[150] Soon DS, Chae MP, Pilgrim CH, Rozen WM, Spychal RT, Hunter-Smith DJ (2016). 3D haptic modelling for preoperative planning of hepatic resection: A systematic review. Ann Med Surg (Lond).

[151] Sugand K, Malik HH, Newman S, Spicer D, Reilly P, Gupte CM (2019). Does using anatomical models improve patient satisfaction in orthopaedic consenting? single-blinded randomised controlled trial. Surgeon.

[152] Sun ML, Zhang Y, Peng Y, Fu DJ, Fan HQ, He R (2020). Accuracy of a novel 3D-printed patient-specific intramedullary guide to control femoral component rotation in total knee arthroplasty. Orthop Surg.

[153] Vaishya R, Patralekh MK, Vaish A, Agarwal AK, Vijay V (2018). Publication trends and knowledge mapping in 3D printing in orthopaedics. J Clin Orthop Trauma.

[154] Wake N, Nussbaum JE, Elias MI, Nikas CV, Bjurlin MA (2020). 3D printing, augmented reality, and virtual reality for the assessment and management of kidney and prostate cancer: a systematic review. Urology.

[155] Wang J, Wang X, Wang B, Xie L, Zheng W, Chen H (2020). Comparison of the feasibility of 3D printing technology in the treatment of pelvic fractures: a systematic review and meta-analysis of randomized controlled trials and prospective comparative studies. Eur J Trauma Emerg Surg.

[156] Wang S, Frisbie J, Keepers Z, Bolten Z, Hevaganinge A, Boctor E (2020). The use of three-dimensional visualization techniques for prostate procedures: a systematic review. Eur Urol Focus.

[157] Witowski JS, Coles-Black J, Zuzak TZ, Pędziwiatr M, Chuen J, Major P (2017). 3D Printing in liver surgery: a systematic review. Telemed J E Health.

[158] Xie L, Chen C, Zhang Y, Zheng W, Chen H, Cai L (2018). Three-dimensional printing assisted ORIF versus conventional ORIF for tibial plateau fractures: A systematic review and meta-analysis. Int J Surg.

[159] Yang JH, Ryu JJ, Nam E, Lee HS, Lee JK (2019). Effects of preoperative virtual reality magnetic resonance imaging on preoperative anxiety in patients undergoing arthroscopic knee surgery: a randomized controlled study. Arthroscopy.

[160] Yang L, Grottkau B, He Z, Ye C (2017). Three dimensional printing technology and materials for treatment of elbow fractures. Int Orthop.

[161] You W, Liu LJ, Chen HX, Xiong JY, Wang DM, Huang JH (2016). Application of 3D printing technology on the treatment of complex proximal humeral fractures (Neer3-part and 4-part) in old people. Orthop Traumatol Surg Res.

[162] Zhang YZ, Chen B, Lu S, Yang Y, Zhao JM, Liu R (2011). Preliminary application of computer-assisted patient-specific acetabular navigational template for total hip arthroplasty in adult single development dysplasia of the hip. Int J Med Robot.

[163] Zhang YZ, Lu S, Zhang HQ, Jin ZM, Zhao JM, Huang J (2016). Alignment of the lower extremity mechanical axis by computer-aided design and application in total knee arthroplasty. Int J Comput Assist Radiol Surg.

[164] Zheng W, Chen C, Zhang C, Tao Z, Cai L (2018). The Feasibility of 3D printing technology on the treatment of pilon fracture and its effect on doctor-patient communication. Biomed Res Int.

[165] Zheng W, Su J, Cai L, Lou Y, Wang J, Guo X (2018). Application of 3D-printing technology in the treatment of humeral intercondylar fractures. Orthop Traumatol Surg Res.

[166] Zheng W, Tao Z, Lou Y, Feng Z, Li H, Cheng L (2018). Comparison of the conventional surgery and the surgery assisted by 3d printing technology in the treatment of calcaneal fractures. J Invest Surg.

