# Supplementary material for: The current and possible future role of 3D modelling within oesophagogastric surgery: a scoping review
Source: Surg Endosc. 2022 Mar 11;36(8):5907–20. doi: 10.1007/s00464-022-09176-z (PMC9283150; doi:10.1007/s00464-022-09176-z)
Supplement: Supplementary file 1 — Supplementary file1 (DOCX 14 KB) [file 464_2022_9176_MOESM1_ESM.docx]

*Appendix 1: Search Strategy for MEDLINE*

| *Search* | *Query* | *Results* |
| --- | --- | --- |
| *1* | *Exp Esophageal Diseases/* | *142020* |
| *2* | *Exp stomach diseases/* | *204194* |
| *3* | *Exp upper gastrointestinal tract/* | *199153* |
| *4* | *Exp duodenal diseases/* | *97861* |
| *5* | *$esophagus.mp.* | *82392* |
| *6* | *Stomach/ or stomach.mp.* | *239092* |
| *7* | *Duodenum.mp.* | *58650* |
| *8* | *Upper gastro*.mp.* | *23904* |
| *9* | *1 or 2 or 3 or 4 or 5 or 6 or 7 or 8* | *537167* |
| *10* | *General surgery.mp. or exp General Surgery/* | *47586* |
| *11* | *Surgery.mp.* | *2632129* |
| *12* | *Surgical.mp.* | *1342309* |
| *13* | *Operation.mp.* | *338843* |
| *14* | *$esophagectomy.mp. or exp esophagectomy/* | *13749* |
| *15* | *Gastrectomy.mp. or exp Gastrectomy/* | *45450* |
| *16* | *10 or 11 or 12 or 13 or 14 or 15* | *3181552* |
| *17* | *9 and 16* | *164788* |
| *18* | *(3D or three dimension*).mp.* | *338859* |
| *19* | *Exp imaging, Three-dimensional/ or volume render.mp.* | *81153* |
| *20* | *18 or 19* | *342110* |
| *21* | *17 and 20* | *791* |
