# Supplementary material for: The current and possible future role of 3D modelling within oesophagogastric surgery: a scoping review
Source: Surg Endosc. 2022 Mar 11;36(8):5907–20. doi: 10.1007/s00464-022-09176-z (PMC9283150; doi:10.1007/s00464-022-09176-z)
Supplement: Supplementary file 2 — Supplementary file2 (DOCX 13 KB) [file 464_2022_9176_MOESM2_ESM.docx]

*Appendix 2: Table of Study Inclusion and Exclusion Criteria*

|  | Inclusion Criteria | Exclusion Criteria |
| --- | --- | --- |
| Population | Adult patients with surgical pathologies of stomach and oesophagus | Non-surgical patients and pathologies, paediatric patients (<16 years old) |
| Context | Oesophagogastric surgical practice and surgical education, all environments considered from lab to theatre | Nil |
| Concept | All forms of 3D reconstruction, 3D modelling and 3D printing applied to surgical practice | 3D reconstruction technologies not applicable to surgical practice |
| Studies | All forms of studies published in peer reviewed literature | Non-human based research |
