# Supplementary material for: The current and possible future role of 3D modelling within oesophagogastric surgery: a scoping review
Source: Surg Endosc. 2022 Mar 11;36(8):5907–20. doi: 10.1007/s00464-022-09176-z (PMC9283150; doi:10.1007/s00464-022-09176-z)
Supplement: Supplementary file 3 — Supplementary file3 (DOCX 28 KB) [file 464_2022_9176_MOESM3_ESM.docx]

*Appendix 3: Characteristics and Themes of All Included Studies*

| Author, Year | Study Design | Origin | Imaging modality | Segmentation method (manual, automatic or semi-automatic) | Rendering (surface vs volume) | Software | Virtual Reality (VR) and/or 3D printing (3DP) | Main theme | Subtheme |
| --- | --- | --- | --- | --- | --- | --- | --- | --- | --- |
| Sankaranarayanan, 2011 | Non-randomised uncontrolled experimental study | USA | - | - | - | Zygote Media Group, Inc. | VR | Surgical education and training | VR simulation |
| Lewis,  2012 | Non-randomised uncontrolled experimental study | UK | - | - | - | Lapmentor VR simulator | VR | Surgical education and training | VR simulation |
| Giannotti,  2014 | Non-randomised uncontrolled experimental study | Italy | - | - | - | Lapmentor VR simulator | VR | Surgical education and training | VR simulation |
| Choi,  2009 | Basic research | South Korea | CT & MR | - | - | Visible Production | VR | Surgical education and training | VR simulation |
| Kavic,  2006 | Basic research | USA | CT | Automatic and manual | Surface rendering | Amira software | VR | Surgical education and training | Anatomical teaching |
| Shin,  2009 | Basic research | South Korea | CT & MR | Automatic and manual | Surface rendering | Alias Maya version 7 (Maya), Rhinoceros | VR | Surgical education and training | Anatomical teaching |
| Kwon,  2015 | Basic research | South Korea | CT & MR | Automatic and manual | Volume rendering | Alias Maya version 7 (Maya), Rhinoceros | VR | Surgical education and training | Anatomical teaching |
| Wu,  2013 | Basic research | China | CT & MR | Manual | Surface and volume rendering | Amira software | VR | Surgical education and training | Anatomical teaching |
| Usui,  2005 | Non-randomised uncontrolled experimental study | Japan | CTA | Automatic | Volume rendering | Zio Software | VR | Operative planning and surgical practice | Pre-operative guidance |
| Matsuo,  2018 | Case report | Japan | CTA | - | - | - | VR | Operative planning and surgical practice | Pre-operative guidance |
| Lee,  2003 | Non-randomised uncontrolled experimental study | Japan | CT | - | Volume rendering | Zio Software | VR | Operative planning and surgical practice | Pre-operative guidance |
| Matsuki,  2004 | Non-randomised controlled experimental study | Japan | CTA | - | Volume rendering | Zio Software | VR | Operative planning and surgical practice | Pre-operative guidance |
| Matsuki,  2006 | Non-randomised controlled experimental study | Japan | CTA | Automatic and manual | Volume rendering | Zio Software | VR | Operative planning and surgical practice | Pre-operative guidance |
| Zheng,  2018 | Case report | China | CT | - | - | - | VR | Operative planning and surgical practice | Pre-operative guidance |
| Li,  2013 | Non-randomised controlled experimental study | China | CTA | - | Volume rendering | TeraRecon | VR | Operative planning and surgical practice | Pre-operative guidance |
| Huang,  2014 | Retrospective Cohort study | China | CT | - | - | - | VR | Operative planning and surgical practice | Pre-operative guidance |
| Wang,  2018 | Non-randomised controlled experimental study | China | CTA | - | Volume rendering | Centricity Radiology | VR | Operative planning and surgical practice | Pre-operative guidance |
| Zhu,  2018 | Retrospective cohort study | South Korea | CTA | Automatic | Volume rendering | VP Planning software | VR | Operative planning and surgical practice | Pre-operative guidance |
| Wu,  2017 | Non-randomised uncontrolled experimental study | China | CTA | - | Volume rendering | PHILIPS Brilliance iCT | VR | Operative planning and surgical practice | Pre-operative guidance |
| Sunagawa and Kinoshita,  2017 | Case Series | Japan | CT | - | Volume rendering | VINCENT software | VR | Operative planning and surgical practice | Pre-operative guidance |
| Peng,  2018 | Case Report | China | CT | - | - | - | VR | Operative planning and surgical practice | Pre-operative guidance |
| Wang,  2014 | Retrospective Case-Control Study | China | CTA | - | - | - | VR | Operative planning and surgical practice | Pre-operative guidance |
| Song,  2019 | Case Report | China | CT | - | - | - | VR | Operative planning and surgical practice | Pre-operative guidance |
| Kinoshita,  2016 | Retrospective cohort study | Japan | CT | Automatic and manual | Volume rendering | VINCENT software | VR | Operative planning and surgical practice | Pre-operative guidance |
| Han,  2014 | Basic Research | South Korea | CTA | Automatic and manual | Volume rendering | Rapidia software | VR | Operative planning and surgical practice | Pre-operative guidance |
| Chang,  2011 | Case Series | South Korea | CT | - | - | 3D-Doctor software | VR | Operative planning and surgical practice | Pre-operative guidance |
| Bosque Lopez,  2010 | Case report | Spain | CT | - | - | - | VR | Operative planning and surgical practice | Pre-operative guidance |
| Wong,  2018 | Case report | Singapore | CT | - | - | - | VR | Operative planning and surgical practice | Pre-operative guidance |
| Chen,  2020 | Retrospective cohort study | China | CT | - | - | 3DSlicer software | VR | Operative planning and surgical practice | Pre-operative guidance |
| Adachi,  2013 | Non-comparative observational study | Japan | CT | - | - | - | VR | Operative planning and surgical practice | Pre-operative guidance |
| Takanami,  2012 | Basic Research | Japan | SPECT/CT & BMIPP | - | - | Zio Software | VR | Operative planning and surgical practice | Pre-operative guidance |
| Kato,  2011 | Basic Research | Japan | CT & MRI | - | - | Zio Software | VR | Operative planning and surgical practice | Pre-operative guidance |
| Disse,  2016 | Prospective Cohort study | France | CT | Manual and semi-automatic | Volume rendering | Vitrea software | VR | Operative planning and surgical practice | Pre-operative guidance |
| Santander,  2019 | Prospective Cohort Study | Germany | CT | Manual and semi-automatic | Volume rendering | TeraRecon software | VR | Operative planning and surgical practice | Pre-operative guidance |
| Huh,  2016 | Basic Research | South Korea | CT | Manual and semi-automatic | Volume rendering | GE medical  software | VR | Operative planning and surgical practice | Pre-operative guidance |
| Cai,  2018 | Non-randomised controlled experimental study | China | CT | - | Surface rendering | Mimics software | VR | Operative planning and surgical practice | Pre-operative guidance |
| Bean,  2005 | Review | USA | CT | - | - |  | VR | Operative planning and surgical practice | Pre-operative guidance |
| Onbaş,  2006 | Non-randomised controlled experimental study | Turkey | CT | - | Volume rendering | Vitrea software | VR | Operative planning and surgical practice | Pre-operative guidance |
| Kim,  2006 | Review | South Korea | CT | - | - | - | VR | Operative planning and surgical practice | Pre-operative guidance |
| Ahn,  2013 | Review | South Korea | CT | - | - | - | VR | Operative planning and surgical practice | Pre-operative guidance |
| Choi,  2014 | Review | South Korea | CT & MRI | - | - | - | VR | Operative planning and surgical practice | Pre-operative guidance |
| Lee,  2010 | Retrospective cohort study | USA | CT | - | Volume rendering | Rapidia software | VR | Operative planning and surgical practice | Pre-operative guidance |
| Mamede,  2007 | Non-randomised controlled experimental study | Japan | PET-CT | Automatic | Volume rendering | MIPAV software | VR | Operative planning and surgical practice | Pre-operative guidance |
| Park,  2010 | Retrospective cohort Study | South Korea | CT | - | - | Rapidia software | VR | Operative planning and surgical practice | Pre-operative guidance |
| Chen,  2007 | Non-randomised controlled experimental study | Taiwan | CT | - | Volume rendering | GE medical software | VR | Operative planning and surgical practice | Pre-operative guidance |
| Kim,  2005 | Review | South Korea | CT | - | - | - | VR | Operative planning and surgical practice | Pre-operative guidance |
| Alfieri,  2015 | Non-randomised controlled experimental study | Italy | CT | Manual, semi-automatic and automatic | Volume rendering | Adaptiva software | VR | Operative planning and surgical practice | Pre-operative guidance |
| Lee,  2007 | Basic Research | South Korea | CT | Semi-automatic | Volume rendering | Rapidia software | VR | Operative planning and surgical practice | Pre-operative guidance |
| Kim,  2005 | Review | South Korea | CT | - | - | - | VR | Operative planning and surgical practice | Pre-operative guidance |
| Singh,  2009 | Review | USA | CT | - | - | - | VR | Operative planning and surgical practice | Pre-operative guidance |
| Duan,  2006 | Non-randomised controlled experimental study | China | CT | - | Volume rendering | SUN magicview  1000 and GE medical software | VR | Operative planning and surgical practice | Pre-operative guidance |
| Marano,  2019 | Case report | Italy | CT | - | - | 3Dific | 3DP and VR | Operative planning and surgical practice | Intra-operative guidance |
| Ye,  2020 | Case report | China | CT | - | - | - | 3DP | Operative planning and surgical practice | Intra-operative guidance |
| Dickinson,  2015 | Case Series | USA | CT | - | - | Mimics  software | 3DP and VR | Operative planning and surgical practice | Intra-operative guidance |
| Sato,  2020 | Case report | Japan | CT | - | - | VINCENT software  &  HoloeyesXR system | VR | Operative planning and surgical practice | Intra-operative guidance |
| Kim,  2013 | Prospective Cohort Study | South Korea | CTA | - | Volume rendering | TeraRecon software, TilePro in da Vinci Surgical system | VR | Operative planning and surgical practice | Intra-operative guidance |
